# Enhanced Co-Expression Extrapolation (COXEN) Gene Selection Method for Building Anti-Cancer Drug Response Prediction Models

**DOI:** 10.3390/genes11091070

**Published:** 2020-09-11

**Authors:** Yitan Zhu, Thomas Brettin, Yvonne A. Evrard, Fangfang Xia, Alexander Partin, Maulik Shukla, Hyunseung Yoo, James H. Doroshow, Rick L. Stevens

**Affiliations:** 1 Computing, Environment and Life Sciences, Argonne National Laboratory, Lemont, IL 60439, USA; brettin@anl.gov (T.B.); fangfang@anl.gov (F.X.); apartin@anl.gov (A.P.); mshukla@anl.gov (M.S.); hsyoo@anl.gov (H.Y.); stevens@anl.gov (R.L.S.); 2 Leidos Biomedical Research, National Laboratory for Cancer Research, Inc. Frederick, Frederick, MD 21702, USA; evrardy@mail.nih.gov; 3 Developmental Therapeutics Branch, National Cancer Institute, Bethesda, MD 20892, USA; doroshoj@mail.nih.gov; 4 Department of Computer Science, The University of Chicago, Chicago, IL 60637, USA

**Keywords:** general drug response prediction model, gene selection, co-expression extrapolation (COXEN), precision oncology

## Abstract

The co-expression extrapolation (COXEN) method has been successfully used in multiple studies to select genes for predicting the response of tumor cells to a specific drug treatment. Here, we enhance the COXEN method to select genes that are predictive of the efficacies of multiple drugs for building general drug response prediction models that are not specific to a particular drug. The enhanced COXEN method first ranks the genes according to their prediction power for each individual drug and then takes a union of top predictive genes of all the drugs, among which the algorithm further selects genes whose co-expression patterns are well preserved between cancer cases for building prediction models. We apply the proposed method on benchmark in vitro drug screening datasets and compare the performance of prediction models built based on the genes selected by the enhanced COXEN method to that of models built on genes selected by the original COXEN method and randomly picked genes. Models built with the enhanced COXEN method always present a statistically significantly improved prediction performance (adjusted *p*-value ≤ 0.05). Our results demonstrate the enhanced COXEN method can dramatically increase the power of gene expression data for predicting drug response.

## 1. Background

Cancer is a heterogeneous disease at both the histologic and genetic levels. Patients with the same cancer histology can respond differently to the same treatment. Accurate prediction of a patient’s response to a drug treatment is of paramount importance to the success of precision oncology. Multiple types of tumor omics data have been used in many studies for predicting anti-cancer drug response [1,2,3,4,5], among which transcriptome data have been shown to be the most important for drug response prediction [6,7]. Because the transcriptome data usually contain the expression values of about 20,000 genes, which can be computationally expensive for training prediction models and easily cause model overfitting on data without a large number of samples, gene selection is frequently applied to select a group of genes most useful for the prediction of drug response [6,8,9].

The co-expression extrapolation (COXEN) method has been developed and used in multiple studies to select genes for predicting the efficacy of a specific drug treatment [8,10,11,12,13,14]. It is designed for applications where the efficacy data of one drug against a set of cancer cases is used to predict the response of another set of cancer cases to the same drug. Gene expression data of both sets of cancer cases are available. The COXEN method identifies the genes predictive of drug response based on the first cancer set. For each predictive gene, the method evaluates how well its co-expression pattern with other predictive genes is preserved between the two sets of cancer cases. Predictive genes that best preserve the co-expression patterns will be used to build a prediction model based on the first cancer set for which drug response is known. The model will then be used to predict drug response on the second cancer set. The COXEN method has been successfully used in multiple situations, for example, building prediction models based on in vitro drug screening data for predicting dug response of other cancer cell lines [8,12], patient tumors [8,14], and even interspecies applications [13].

However, a limitation of the original COXEN method is that it is designed for selecting genes that are predictive of the efficacy of one drug. In many studies, it is necessary to build general drug response prediction models that can be trained on the efficacy data of multiple drugs and that are not specific to a particular drug of interest [1,5,15,16]. In general drug response prediction models, drugs can be represented by molecular descriptors, fingerprints, SMILES strings, and others [1,5,15,16]. The benefits of building general prediction models include an increased size of training data by combining the efficacy data of multiple drugs and the ability of predicting the response of multiple drug treatments and even new drugs that have not been tested before. Because drugs can have very different mechanisms of actions, genes that are predictive of the efficacy of one drug may not be useful for predicting the efficacy of another drug. Therefore, the original COXEN method is not ideal for building general drug response prediction models.

To meet this challenge, we propose an enhanced COXEN method that extends the original method to multi-drug cases. Rather than selecting genes predictive of the response to one drug, the enhanced COXEN method selects genes that are predictive of multiple drug activities. It first ranks the genes according to their prediction power for each individual drug. Then, the same number of top predictive genes are selected for every drug and their union is taken to form a candidate pool, based on which the preservation of co-expression pattern will be considered to further select genes. We test the proposed enhanced COXEN method on three benchmark in vitro drug screening datasets. General drug response prediction models are built based on the selected genes and their prediction performances are evaluated and compared to those of models constructed based on randomly picked genes and genes selected by the original COXEN method. The results demonstrate that genes selected by the enhanced COXEN method always provide a statistically significantly improved prediction performance (adjusted *p*-value ≤ 0.05) and increase the power of gene expression data for drug response prediction.

## 2. Methods

**Figure 1:**
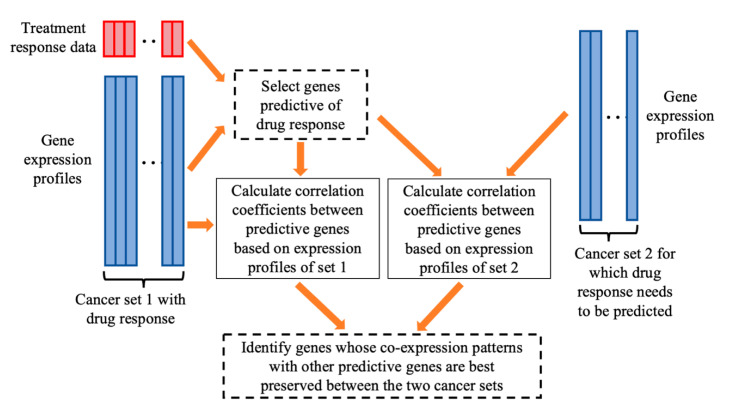
Analysis flowchart of the COXEN framework. The original COXEN method and the enhanced COXEN method are different in the two boxes with dashed line border.

In this section, we will first review the original COXEN method [8,10] and then enhance it for the general, multi-drug prediction task. Figure 1 illustrates the analysis flowchart of the COXEN framework, in which the original COXEN method and the enhanced COXEN method differ in the two boxes indicated by dashed line border. The original COXEN method starts with the gene expression profiles of two sets of cancer cases to select a group of predictive and generalizable genes for predicting drug response. The response of the first cancer set to a drug treatment was measured, and the response of the second set to the same drug needs to be predicted. Table 1 gives the details of the original COXEN method that takes three major steps. Step 1 uses a prediction power measure (PPM), such as the absolute value of the Pearson correlation coefficient between gene expression and treatment response, to measure each gene’s power for predicting the drug response. Top predictive genes are then selected to form a candidate pool. Step 2 calculates a generalization score for each gene in the candidate pool, which evaluates how well a gene’s co-expression pattern with all other candidate genes is preserved between the two sets of cancer cases. Step 3 further selects a subset of genes from the candidate pool whose co-expression patterns are best preserved for building prediction models. Gene co-expression patterns characterize the transcriptional regulation mechanisms between genes. A higher generalization score of a gene indicates that the transcriptional regulation relationship between the gene and the rest of the predictive genes is better preserved. Thus, genes with high scores may be more generalizable for predicting drug response on new cancer cases. Through these three steps, the COXEN method considers both prediction power and generalizability of genes for building the prediction model. After gene selection, a prediction model will be trained on the first set of cancer cases based on the selected genes, and then used to predict the drug response of the second set.

**Figure 2:**
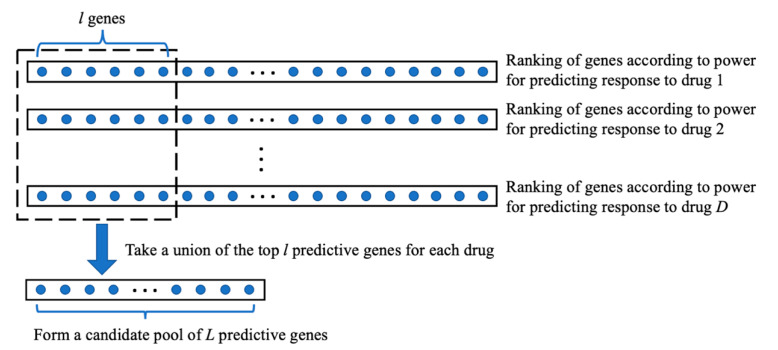
An illustration of generating the candidate gene pool by taking a union of top predictive genes for each drug.

In the multi-drug case, treatment responses to multiple drugs are available in dataset 1, although it is not necessary for all cancer cases to have been treated by all drugs. We propose to enhance Step 1 in the COXEN method for selecting genes that can be used to predict the responses to multiple drugs. For each drug, the enhanced scheme extracts the gene expression profiles of cancer cases treated by the drug and the corresponding drug response values. Each gene’s power for predicting the drug response is calculated using a defined PPM and all genes are ranked according to their prediction power. The calculation of prediction power and ranking of genes are conducted for all drugs. Then, a union of the top predictive genes of all the drugs is taken to form a candidate pool. See Figure 2 for an illustration. The same number (denoted by *l*) of top predictive genes are selected for each drug. In the algorithm, *l* starts at ceil (*N*_1_/*D*), where *D* is the number of drugs and ceil (·) calculates the smallest integer not smaller than the input. The union of the top *l* predictive genes of all *D* drugs is calculated, whose size is denoted by *L*. If *L* is smaller than *N*_1_, *l* is increased by one and the gene set union is recalculated. The increasing of *l* by one is repeated until *L* becomes no smaller than *N*_1_. Then the *L* genes form the candidate predictive gene pool, from which $N_{2}$ generalizable genes will be further selected.

Besides extending the selection of predictive genes to the multi-drug case by modifying Step 1, we also improve the generalizable gene selection (Step 2 of the COXEN method) by replacing the Pearson correlation coefficient with the concordance correlation coefficient (CCC) [17] for calculating the generalization score in Step 2.3, which is also suggested by the recent study conducted by Kim et al. [9]. CCC is calculated by the following formula,

(1) $$\mathrm{CCC}\left(c_{1},c_{2}\right)=\frac{2\mathrm{cov}\left(c_{1},c_{2}\right)}{\mathrm{var}\left(c_{1}\right)+\mathrm{var}\left(c_{2}\right)+{\left(\bar{c_{1}}-\bar{c_{2}}\right)}^{2}}$$

where cov(·, ·) is the covariance of two variables, var(·) is the variance of a variable, and $\bar{c_{1}}$ and $\bar{c_{2}}$ indicates the mean of $c_{1}$ and $c_{2}$, respectively. Unlike the Pearson correlation coefficient that evaluates the linear correlation between two variables, the concordance correlation coefficient evaluates how much two variables deviate from the main diagonal (45° line) in the scatter plot, which more stringently evaluates the consistency between two variables, and thus better evaluates the preservation of co-expression patterns. CCC takes a value in [−1, 1]. It is 1 if and only if $c_{1}$ and $c_{2}$ are exactly identical; it is −1 if and only if $c_{1}$ and $c_{2}$ reversely match; and it is 0 if and only if $c_{1}$ and $c_{2}$ are uncorrelated.

## 3. Results

**Figure 3:**
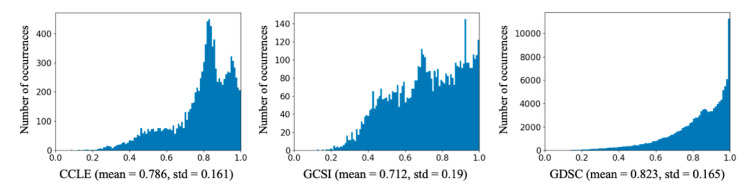
Histograms of drug response AUC values in datasets. Mean and standard deviation (std) of AUC values are shown under each histogram.

To evaluate the proposed enhanced COXEN method, we applied it on three benchmark in vitro drug screening datasets, the Cancer Cell Line Encyclopedia (CCLE) dataset [18], the Genentech Cell Line Screening Initiative (GCSI) dataset [19], and the Genomics of Drug Sensitivity in Cancer (GDSC) dataset [20]. See Table 2 for the numbers of cancer cell lines (CCLs), drugs, and experiments (pairs of drugs and CCLs) in the three datasets. We used the three-parameter sigmoid function (hill slope model) to fit the tumor cell growth values and generate dose response curves. Based on the dose response curve, we calculated the area under the dose response curve (AUC) for the dose range of [10^−10^ M, 10^−4^ M]. The AUC value was then normalized by the dose range, so that after normalization the AUC value was between 0 and 1, where 0 indicated complete response and 1 indicated no response. The AUC value was the prediction target in our analysis. See Figure 3 for the distributions of AUC value in the datasets. The RNA-seq gene expression data of CCLs were obtained from the CCLE online resource. Although GDSC and GCSI do not provide RNA-seq data, almost all the CCLs used in the two studies were also included in the CCLE study. Thus, we used their RNA-seq data from the CCLE resource. There were 11 GDSC CCLs not included in the CCLE resource, which were excluded from the analysis. We removed the genes whose average expression values across cell lines were the smallest, and 15,732 genes were kept for analysis. The drugs were represented by numeric descriptors calculated using the Dragon (version 7.0) software package (https://chm.kode-solutions.net/products_dragon.php) based on their molecular structures. A total of 1623 drug descriptors that were not missing in any drug were used for analysis.

Based on the genes selected by the enhanced COXEN method, we used LightGBM [21], a representative and efficient gradient boosting algorithm, to build regression models for predicting the AUC value. The mean squared error was used as the loss function for model training. Expression values of the selected genes and the drug descriptors were concatenated to form the input data for model training. On the two small datasets, CCLE and GCSI, the model training process would be stopped early if the validation loss did not reduce in 150 boosting steps; otherwise, the full training process would take 1500 steps. On the large dataset GDSC, we increased the number of training steps, where the full training process would take 5000 steps and it would stop early if the validation loss did not reduce in 500 steps. We used the LightGBM Python package (https://LightGBM.readthedocs.io/en/latest/index.html) to implement the analysis pipeline and all other parameters took the default values. Coefficient of determination (R^2^) was used to evaluate the prediction performance. For a reliable evaluation of the prediction performance, 10-fold cross-validation was conducted 10 times, thus including 100 cross-validation trials. In a cross-validation trial, a dataset is divided into 10 data folds that do not share CCLs. Eight data folds were used for model training: one data fold was used for validation to stop the training early, and the rest were used for testing. Because the data folds did not share CCLs, such a cross-validation scheme simulated precision oncology applications that need to predict the response of new cancer cases to existing drugs.

We compared the prediction performance of models built based on genes selected by the enhanced COXEN method to those of prediction models built based on three baseline gene selection methods, including genes randomly selected from all available genes, genes randomly selected from the LINCS gene set that includes 942 “landmark” genes that well represent cellular transcriptomic changes identified in the Library of Integrated Network-Based Cellular Signatures (LINCS) project [22], and genes selected by the original COXEN method that was applied ignoring the drug difference. The performance of the baseline methods was also evaluated through 100 10-fold cross-validation trials with the same data partitions as used in the evaluation of the enhanced COXEN method. In each cross-validation trial, the training set was taken as dataset 1 and the validation set and the testing set were combined to be dataset 2, in order to apply the enhanced and original COXEN methods. The absolute value of Pearson correlation coefficient between a gene’s expression values and the drug response values was used to measure the gene’s prediction power in both the enhanced and original COXEN methods. For $N_{1}$ and $N_{2}$ in the enhanced and original COXEN methods, we tried 1600, 800, 400, 200 and 800, 400, 200, 100, respectively. Note that the number of genes selected by the enhanced COXEN method was always equal to the number of genes selected by the baseline methods for a fair comparison.

Although R^2^ is good for measuring the prediction performance of regression models, it is not enough to accurately characterize the influence of gene selection method on the expression data for drug response prediction. The reason is that in multi-drug cases, the drug difference contributes much more to the drug response variation than the tumor difference does. This can be examined by calculating and comparing the between-class variation and within-class validation while taking drugs and CCLs as classes in the data. The following formulas give the definitions of between-class variation, within-class variation, and total variation

(2) $$S_{t}=\overset{K}{\underset{k=1}{\sum }}\overset{N_{k}}{\underset{i=1}{\sum }}{\left(r_{i}-\bar{m}\right)}^{2}$$

(3) $$S_{b}=\overset{K}{\underset{k=1}{\sum }}N_{k}{\left({\bar{m}}_{k}-\bar{m}\right)}^{2}$$

(4) $$\;S_{w}=\overset{K}{\underset{k=1}{\sum }}\overset{N_{k}}{\underset{i=1}{\sum }}{\left(r_{i}-{\bar{m}}_{k}\right)}^{2}$$

(5) $$\bar{m}=\frac{1}{\sum_{k=1}^{K}N_{k}}\overset{K}{\underset{k=1}{\sum }}\overset{N_{k}}{\underset{i=1}{\sum }}r_{i}\;$$

(6) $${\bar{m}}_{k}=\frac{1}{N_{k}}\overset{N_{k}}{\underset{i=1}{\sum }}r_{i}$$

where $S_{b}$, $S_{w}$, and $S_{t}$ are between-class, within-class, and total variations, respectively, K is the number of classes (i.e., the number of drugs if drugs are taken as classes or the number of CCLs if CCLs are taken as classes), $N_{k}$ is the number of experiments in class *k*, $r_{i}$ is the drug response value of the *i*th experiment in class *k*, $\bar{m}$ is the average drug response value of all experiments, ${\bar{m}}_{k}$ is the average drug response value of experiments in class *k*. Notice that $S_{t}=S_{b}+S_{w}$. We calculated the between-class, within-class, and total variations on the datasets taking CCLs and drugs as classes (see Table 3). On the CCLE dataset, the between-class variation is 192.60/21.93 = 8.78 folds higher when drugs are taken as classes than when CCLs are taken as classes. This ratio is 5.48 and 8.96 for the GCSI and GDSC datasets, respectively. Because the drug difference dominates the drug response variation, the overall prediction performance (R^2^) is mainly contributed by the drug descriptor data that well represent the drug difference, while the gene expression data of CCLs have a relatively small contribution. Thus, the overall prediction performance is not ideal for characterizing how the gene selection method influences the prediction power of gene expression data alone. To meet this need, we calculated the following performance improvement percentage (PIP) measure:

(7) $$\mathrm{PIP}=\frac{\frac{1}{100}\sum_{t=1}^{100}R_{\mathrm{EnhancedCOXEN},t}^{2}-\frac{1}{100}\sum_{t=1}^{100}R_{\mathrm{Baseline},t}^{2}}{\frac{1}{100}\sum_{t=1}^{100}R_{\mathrm{Baseline},t}^{2}-\frac{1}{100}\sum_{t=1}^{100}R_{\mathrm{DD},t}^{2}}\times 100\%$$

where *t* is the index of cross-validation trial, $R_{\mathrm{DD},t}^{2}$ is the prediction performance (R^2^) of a model built based on only drug descriptors without any gene expression data involved for cross-validation trial *t*, $R_{\mathrm{Baseline},t}^{2}$ is the prediction performance of the model built based on drug descriptors and expressions of genes selected by a baseline method for cross-validation trial *t*, and $R_{\mathrm{EnhancedCOXEN},t}^{2}$ is the prediction performance of the model built based on drug descriptors and expressions of genes selected by the enhanced COXEN method for cross-validation trial *t*. PIP calculates the percentage of performance improvement of the enhanced COXEN method on the basis of the “pure” contribution of the baseline gene selection method in prediction performance. It evaluates how much the enhanced COXEN method improves the prediction power of gene expression data compared to the baseline method. The average of $R_{\mathrm{DD},t}^{2}$ across 100 cross-validation trials was 0.678, 0.600, and 0.570 for the CCLE, GCSI, and GDSC datasets, respectively.

Table 4 shows the comparison of the prediction performance of models built based on genes selected by the enhanced COXEN method and that of models built based on genes selected by the baseline gene selection methods. For all three datasets, and with all different numbers of selected genes, models built based on genes selected by the enhanced COXEN method always outperformed models built based on genes randomly selected from all available genes, as demonstrated by the increased average R^2^. Pairwise t-tests with multiple test correction using the Benjamini-Hochberg (BH) procedure [23] indicate that this performance improvement is also always statistically significant (adjusted *p*-values ≤ 0.05). The PIP ranges from 11.7% to 43.5% with an average of 25.1%, indicating that the enhanced COXEN method dramatically improves the prediction power of gene expression data compared to genes randomly picked from all available genes. Compared between different numbers of selected genes ($N_{2}$) on each dataset, the PIP shows an increasing trend when fewer genes are used for drug response prediction. This indicates the importance of using the enhanced COXEN method to select genes when the prediction model uses a limited number of input genes.

In the comparison between the enhanced COXEN gene selection and random genes picked from the LINCS gene set [22], again, models built using the enhanced COXEN method always statistically significantly outperformed models built using randomly picked genes (adjusted *p*-values ≤ 0.05), evaluated by the average R^2^ across cross-validation trials and pair-wise t-tests adjusted using the BH procedure (Table 4). The PIP ranges from 12.2% to 37.2%, with an average of 21.9%, indicating a dramatic improvement on the prediction power of gene expression data by using the enhanced COXEN gene selection. The results also show a general increasing trend in PIP when smaller numbers of genes are used for drug response prediction.

In the comparison between the enhanced and original COXEN methods, the enhanced COXEN method always statistically significantly outperformed the original COXEN method (adjusted *p*-values ≤ 0.05), evaluated by the average R^2^ across cross-validation trials and pair-wise t-tests adjusted using the BH procedure (Table 4). The PIP, which indicates how much the enhanced COXEN method improves the prediction power of gene expression data compared to the original COXEN method, has a range of 14.8–17.9%, 6.3–13.4%, and 3.0–5.6% on the CCLE, GDSC, and GCSI datasets, respectively. Apparently, PIPs obtained on the CCLE and GDSC datasets are higher than that obtained on the GCSI dataset. A potential reason may be that the drug difference more severely dominates the drug response variation in the CCLE and GDSC datasets than in the GCSI dataset, as shown above. When the drug difference dominates the drug response variation, the original COXEN method may tend to select genes whose expression values correlated with the change of treatment response between drugs, which might not bring additional prediction power as the drug descriptors may already well characterize the difference between drugs. The enhanced COXEN method selects predictive genes for each individual drug based on the gene expression profiles of cancer cases treated by the drug and the corresponding drug response values. Thus, the selection of predictive genes in the enhanced COXEN method is not affected by the variation of treatment response between drugs, and tends to select genes correlated with the response change between different cancer cases treated by a drug, which may better help improve the prediction performance upon drug descriptors.

To examine the performance of general drug response prediction models not specific to a drug, it is certainly important to calculate the overall prediction performance across drugs, as we have done above. However, it is also interesting to compare the gene selection methods for their influence on predicting the response to individual drugs. Notice that such an evaluation will ignore the contribution of both gene expressions and drug descriptors for predicting the response variation between drugs. On each dataset and for each drug, we calculated the R^2^ of predicting the response of the CCLs treated by the drug for both the enhanced COXEN method and the three baseline gene selection methods. The average and standard deviation of R^2^ across drugs are calculated and shown in Table 5. The average R^2^ of the enhanced COXEN method for predicting the response to individual drugs is always higher than that of the baseline gene selection methods. Pairwise t-test is also applied across the drugs to evaluate the statistical significance of this performance improvement, with the *p*-values corrected for multiple tests using the BH procedure. On the large dataset GDSC, with more drugs, the performance improvement was always statistically significant (adjusted *p*-values ≤ 0.05). On the two smaller datasets, CCLE and GCSI, it sometimes did not reach the cutoff to be deemed statistically significant, partially due to the relatively small numbers of drugs screened in the studies providing insufficient sample sizes to reach the significance cutoff.

Aside from the original COXEN method and randomly selected genes, we also compared the enhanced COXEN method to a set of hub genes derived from co-expression network analysis that are predictive of drug response [24]. Azuaje et al. performed a pan-cancer co-expression network analysis based on more than 1000 CCLs, and identified 47 genes as representing “hubs” in the network [24]. A total of 45 out of the 47 genes were included in the gene expression data used in our analysis. We built general drug response prediction models using LightGBM based on the 45 genes, and compared their prediction performance to that of models built based on genes selected by the enhanced COXEN method. For a fair comparison, the enhanced COXEN method was applied to select 45 genes. We performed 10 10-fold cross-validations (totally 100 cross-validation trials) using the same data partitions as used in the analyses above. The prediction performance R^2^ was calculated for each cross-validation trail and the pair-wise t-test was conducted to evaluate the significance of performance difference between the enhanced COXEN method and the hub genes, with *p*-values corrected for multiple tests using the BH procedure. Table 6 shows the obtained results. On the two small datasets, CCLE and GCSI, the enhanced COXEN method showed a statistically significantly improved R^2^ (adjusted *p*-values ≤ 0.05), with PIP values of 25.2% and 23.3%, respectively. On the large dataset GDSC with 238 drugs, the enhanced COXEN method had almost the same prediction performance as the hub genes, with no statistically significant difference. A potential reason is that compared to the diversity of drugs in the GDSC dataset, too few genes were selected to build prediction models, and they might not be sufficient to model the activities of all the drugs. Thus, with the limited number of genes selected, the enhanced COXEN method was unable to demonstrate its advantage.

## 4. Conclusions

We developed an enhanced COXEN method to select predictive and generalizable genes for building general drug response prediction models. The method selects genes that are predictive of the efficacies of multiple drugs simultaneously, and whose co-expression patterns are preserved between cancer cases. It ranks the genes according to their prediction power for each individual drug and then takes a union of the same number of top predictive genes for all the drugs. Co-expression patterns between the predictive genes are evaluated by the Pearson correlation coefficients, based on which the concordance correlation coefficient is used to evaluate the preservation of co-expression patterns between cancer cases. We tested the enhanced COXEN method on three in vitro drug screening datasets. Models constructed using the genes selected by the enhanced COXEN method always showed a prediction performance statistically significantly (adjusted *p*-values ≤ 0.05) better than that of models built using genes identified by the original COXEN method and genes randomly picked from either all available genes or the representative LINCS gene set. The results demonstrate that the enhanced COXEN method dramatically increases the power of gene expression data for drug response prediction. Compared to the original COXEN method, the advantage of the enhanced COXEN method is more pronounced on datasets in which the drug response variation is more dominated by drug diversity.

## Figures and Tables

**Table 1:** The original COXEN gene selection method.

Given:	Dataset 1 that includes an $P\times M_{1}$ matrix of gene expressions for *P* genes and $M_{1}$ cancer cases and the response values of the cancer cases to the treatment of a drug. Dataset 2, an $P\times M_{2}$ matrix of gene expressions for $M_{2}$ cancer cases, for which we want to predict their sensitivities to the drug treatment. Two positive integers $N_{1}$ and $N_{2}$, $N_{1}$ > $N_{2}$ > 1.
Step 1:	On dataset 1, for every gene, calculate the gene’s prediction power for drug response using a defined PPM. Select $N_{1}$ genes with the strongest prediction power.
Step 2:	For each of the $N_{1}$ genes, do the following. Step 2.1: Calculate its Pearson correlation coefficients with the other $N_{1}-1$ genes based on their expression values in dataset 1, which forms an $N_{1}-1$ dimensional vector denoted by $c_{1}$ Step 2.2: Calculate its Pearson correlation coefficients with the other $N_{1}-1$ genes based on their expression values in dataset 2, forming another $N_{1}-1$ dimensional vector denoted by $c_{2}$ Step 2.3: Calculate the Pearson correlation coefficient between $c_{1}$ and $c_{2}$, which forms the generalization score of the gene.
Step 3:	Among the $N_{1}$ genes, select $N_{2}$ genes with the highest generalization scores, which will be used for building a prediction model of drug response.

**Table 2:** Numbers of CCLs, drugs, and experiments (pairs of drugs and CCLs) in datasets.

Dataset	# CCLs	# Drugs	# Experiments
GCSI	357	16	5647
CCLE	474	24	10,971
GDSC	659	238	125,712

**Table 3:** Comparison of between-class and within-class drug response variations with CCLs and drugs taken as classes.

Dataset	Total Variation	Between-Class Variation (CCL)	Within-Class Variation (CCL)	Between-Class Variation (Drug)	Within-Class Variation (Drug)
CCLE	282.97	21.93	261.05	192.60	90.37
GCSI	203.14	22.43	180.71	122.85	80.30
GDSC	3413.74	218.11	3195.64	1954.38	1459.37

**Table 4:** Comparison on the prediction performance of models built based on genes selected by the enhanced COXEN method and that of models built based on genes selected by the baseline gene selection methods.

Data	*N*_1_	*N*_2_	Enhanced COXEN	Random All			Random LINCS			Original COXEN		
			R^2^	R^2^	Adjusted *p*-Value	PIP	R^2^	Adjusted *p*-Value	PIP	R^2^	Adjusted *p*-Value	PIP
CCLE	1600	800	0.725 (0.018)	0.715 (0.019)	4.32 × 10^−25^	27.3%	0.716 (0.018)	1.57 × 10^−24^	23.0%	0.719 (0.018)	2.58 × 10^−19^	16.0%
	800	400	0.724 (0.018)	0.712 (0.019)	7.04 × 10^−30^	33.5%	0.714 (0.018)	8.04 × 10^−24^	26.5%	0.717 (0.018)	1.36 × 10^−15^	16.3%
	400	200	0.721 (0.019)	0.710 (0.019)	5.74 × 10^−24^	33.5%	0.711 (0.018)	9.66 × 10^−20^	29.9%	0.715 (0.019)	2.81 × 10^−11^	14.8%
	200	100	0.719 (0.019)	0.706 (0.019)	6.23 × 10^−20^	43.5%	0.708 (0.020)	1.74 × 10^−18^	37.2%	0.713 (0.018)	2.81 × 10^−10^	17.9%
GCSI	1600	800	0.678 (0.032)	0.670 (0.031)	1.03 × 10^−10^	11.7%	0.669 (0.032)	2.60 × 10^−13^	14.4%	0.676 (0.032)	4.10 × 10^−3^	3.4%
	800	400	0.678 (0.034)	0.666 (0.032)	6.64 × 10^−13^	18.0%	0.666 (0.033)	1.54 × 10^−13^	18.0%	0.675 (0.031)	2.21 × 10^−2^	3.0%
	400	200	0.675 (0.036)	0.661 (0.033)	3.97 × 10^−13^	23.8%	0.661 (0.032)	2.60 × 10^−13^	22.7%	0.672 (0.033)	1.62 × 10^−2^	4.0%
	200	100	0.672 (0.036)	0.653 (0.033)	1.59 × 10^−15^	35.1%	0.654 (0.033)	9.29 × 10^−18^	32.1%	0.668 (0.032)	1.00 × 10^−2^	5.6%
GDSC	1600	800	0.625 (0.017)	0.618 (0.017)	7.40 × 10^−24^	15.0%	0.619 (0.017)	1.64 × 10^−20^	12.2%	0.621 (0.017)	9.52 × 10^−12^	6.3%
	800	400	0.624 (0.018)	0.616 (0.017)	2.35 × 10^−20^	15.5%	0.617 (0.017)	4.99 × 10^−18^	13.0%	0.619 (0.018)	3.86 × 10^−12^	8.4%
	400	200	0.622 (0.017)	0.614 (0.018)	3.95 × 10^−25^	19.9%	0.615 (0.018)	1.35 × 10^−18^	15.3%	0.617 (0.017)	3.55 × 10^−16^	11.1%
	200	100	0.620 (0.018)	0.610 (0.018)	4.65 × 10^−25^	24.7%	0.612 (0.018)	1.76 × 10^−15^	18.1%	0.614 (0.017)	5.21 × 10^−19^	13.4%

Enhanced COXEN, Original COXEN, Random All, and Random LINCS refer to the enhanced COXEN method, the original COXEN method, genes randomly picked from all available genes, and genes randomly picked from the LINCS set, respectively. In the R^2^ columns, the number before the parenthesis is the average R^2^ across cross-validation trails and the number in the parenthesis is the standard deviation.

**Table 5:** Comparison of the enhanced COXEN method and the baseline gene selection methods in the performance of predicting response to individual drugs.

Data	*N*_1_	*N*_2_	Enhanced COXEN	Random All		Random LINCS		Original COXEN	
			R^2^	R^2^	Adjusted *p*-Value	R^2^	Adjusted *p*-Value	R^2^	Adjusted *p*-Value
CCLE	1600	800	0.160 (0.110)	0.147 (0.100)	2.63 × 10^−3^	0.139 (0.091)	4.72 × 10^−4^	0.147 (0.103)	1.66 × 10^−2^
	800	400	0.153 (0.110)	0.143 (0.096)	8.80 × 10^−2^	0.136 (0.091)	7.26 × 10^−3^	0.144 (0.103)	1.68 × 10^−1^
	400	200	0.141 (0.109)	0.135 (0.093)	2.85 × 10^−1^	0.133 (0.087)	1.80 × 10^−1^	0.138 (0.102)	7.42 × 10^−1^
	200	100	0.130 (0.105)	0.126 (0.088)	5.21 × 10^−1^	0.129 (0.087)	9.21 × 10^−1^	0.129 (0.097)	7.98 × 10^−1^
GCSI	1600	800	0.222 (0.103)	0.215 (0.104)	3.05 × 10^−1^	0.207 (0.102)	1.93 × 10^−2^	0.213 (0.101)	2.50 × 10^−1^
	800	400	0.219 (0.107)	0.206 (0.100)	1.10 × 10^−1^	0.206 (0.100)	4.97 × 10^−2^	0.216 (0.102)	7.42 × 10^−1^
	400	200	0.208 (0.107)	0.198 (0.096)	2.30 × 10^−1^	0.190 (0.097)	1.93 × 10^−2^	0.205 (0.100)	7.42 × 10^−1^
	200	100	0.203 (0.101)	0.181 (0.095)	4.16 × 10^−2^	0.178 (0.092)	1.03 × 10^−2^	0.195 (0.095)	5.34 × 10^−1^
GDSC	1600	800	0.085 (0.118)	0.074 (0.113)	2.99 × 10^−25^	0.073 (0.116)	1.78 × 10^−38^	0.080 (0.116)	1.37 × 10^−11^
	800	400	0.083 (0.117)	0.074 (0.111)	9.36 × 10^−21^	0.073 (0.113)	4.54 × 10^−26^	0.077 (0.114)	1.38 × 10^−10^
	400	200	0.083 (0.115)	0.070 (0.110)	4.26 × 10^−26^	0.072 (0.113)	4.92 × 10^−22^	0.075 (0.114)	5.24 × 10^−14^
	200	100	0.080 (0.114)	0.067 (0.105)	1.14 × 10^−20^	0.069 (0.109)	4.62 × 10^−22^	0.071 (0.113)	3.70 × 10^−17^

Enhanced COXEN, Original COXEN, Random All, and Random LINCS refer to the enhanced COXEN method, the original COXEN method, genes randomly picked from all available genes, and genes randomly picked from the LINCS set, respectively. In the R^2^ columns, the number before the parenthesis is the average R^2^ across drugs and the number in the parenthesis is the standard deviation.

**Table 6:** Comparison in the performance of prediction models built based on genes selected by the enhanced COXEN method and that of models built based on the hub genes derived from network analysis.

Data	*N*_1_	*N*_2_	R^2^ (Enhanced COXEN)	R^2^ (Hub Genes)	Adjusted *p*-Value	PIP
CCLE	90	45	0.716 (0.019)	0.709 (0.020)	1.14 × 10^−^^10^	25.2%
GCSI	90	45	0.668 (0.035)	0.655 (0.033)	5.07× 10^−^^9^	23.3%
GDSC	90	45	0.616 (0.018)	0.617 (0.017)	6.75× 10^−^^1^	−0.7%

Enhanced COXEN and Hub Genes refer to the enhanced COXEN method and the hub genes identified from co-expression network analysis [24], respectively. In the R^2^ columns, the number before the parenthesis is the average R^2^ across cross-validation trails and the number in the parenthesis is the standard deviation.

## Abbreviations

COXEN: Co-expression extrapolation; PPM: Prediction power measure; CCC: Concordance correlation coefficient; CCL: Cancer Cell Line; CCLE: Cancer Cell Line Encyclopedia; GCSI: Genentech Cell Line Screening Initiative; GDSC: Genomics of Drug Sensitivity in Cancer; AUC: Area under the dose response curve; R^2^: Coefficient of determination; LINCS: Library of Integrated Network-Based Cellular Signatures; PIP: Performance improvement percentage.
